# Platelet-Rich Fibrin: A Self-Derived Biomaterial for Surgical Treatment of a Periapical Lesion

**DOI:** 10.7759/cureus.64796

**Published:** 2024-07-18

**Authors:** Yash Sinha, Debangana Bhattacharjee, Prasanti Pradhan, Akansha Tilokani, Aanchal Banka

**Affiliations:** 1 Department of Conservative Dentistry and Endodontics, Kalinga Institute of Dental Sciences, Kalinga Institute of Industrial Technology (KIIT) Deemed to be University, Bhubaneswar, IND

**Keywords:** platelet-rich fibrin, resin-based sealer, calcium hydroxide, biodentine, prf, periapical lesion

## Abstract

This case report explores the application of platelet-rich fibrin (PRF) as an autologous biomaterial in the surgical management of a periapical cyst in an 18-year-old female patient. The patient presented with pain, discoloration, and swelling in the maxillary left central incisor region, indicative of an asymptomatic periapical lesion associated with a history of trauma. Despite initial endodontic treatment with calcium hydroxide, the lesion persisted, necessitating surgical intervention.

PRF, prepared from the patient’s blood, was utilized during periapical surgery to promote healing and tissue regeneration. The surgical procedure included enucleation of the cyst, apicoectomy, and retrograde filling with Biodentine. Clinical and radiographic assessments at follow-up visits (three, six, and nine months post-surgery) revealed successful healing with no signs of inflammation or discomfort.

The use of PRF demonstrated favorable outcomes in enhancing wound healing and maintaining a favorable environment for tissue remodeling. This case underscores the potential of PRF as an effective biomaterial in periapical surgery, advocating for its integration into dental therapeutic strategies for its regenerative properties and cost-effectiveness.

## Introduction

Infections in both the intra- and extraradicular domains are invariably linked to cyst-like lesions [1]. These lesions appear to be growing larger on radiographs but do not exhibit any painful symptoms [2]. When there is a consistent alteration in the periradicular tissue that is unresponsive to nonsurgical treatment, surgery is frequently the preferred course of action. Platelet-rich fibrin (PRF) forms a fibrin clot by aggregating platelets and the released cytokines. As platelet cytokines in the PRF are important mediators of inflammation and rapid wound healing [3], measuring them is an important step in the procedure. PRF exhibits multiple vital features, including cell attachment, migration, multiplication, and differentiation. In fact, it contains platelet-derived growth factor, transforming growth factor β1, insulin-like growth factor, vascular endothelial growth factor, and other growth factors [4]. PRF prevents undesirable cells from invading too soon and serves as an interpositional material and a powerful, effective barrier between wanted and unwanted cells. When used as a palliative biomaterial, it encourages mucous membrane healing and accelerates wound closure by liberating fibrin dressing and certain growth factors. [5]. Furthermore, it was once thought to be a strong biomaterial for pulp-dentin complex regeneration [6]. PRF preparation is a simple and low-cost process. By removing the unwarranted step of adding bovine thrombin to platelet-rich plasma, PRF encourages the transformation of fibrinogen to fibrin.

## Case presentation

An 18-year-old female patient reported to the Department of Conservative Dentistry and Endodontics, Kalinga Institute of Dental Sciences, Kalinga Institute of Industrial Technology, Bhubaneswar, Odisha with a complaint of pain, discolored tooth, and mild swelling in the upper front tooth region since 15 days with a history of trauma 10 years ago. On visual examination, discoloration was observed with respect to the maxillary left central incisor (21), which was asymptomatic and revealed no response on pulp sensibility testing. Periapical radiolucency with respect to 21 was revealed by radiographic examination. The lesion’s diameter labio-palatally and mesio-distally was 8 mm and 5 mm, respectively, with labial cortical plate perforation, according to cone-beam CT findings.

Endodontic treatment protocol

After the completion of the access opening with respect to 21, an apex locator (Root ZX Mini, J Morita, Japan) was used to measure its working length and was verified by obtaining a radiograph. A K-file was used for the shaping and cleaning. The #50K-file was established as the master apical file, and the #80K-file was applied using the step-back technique. During the biomechanical preparation, 5.25% sodium hypochlorite and 17% EDTA gel (Anabond Endoprep-Rc) were used as the irrigants simultaneously, with saline being the final irrigating solution. Although calcium hydroxide (RC Cal, Prime Dental, Mumbai, India) was used twice at one-week intervals as an intracanal medicament, the patient’s symptoms persisted. Hence, periapical surgery was planned after obturation by lateral condensation technique using AH plus sealer and gutta-percha points.

Preparation of platelet-rich fibrin

During the procedure, the patient had about 10 mL of venous blood drawn. This blood was collected in two sterile, 5-mL vacutainer tubes without the use of an anticoagulant. These tubes were run through a tabletop centrifugation machine for nearly 10 minutes at a constant speed of 3,000 rpm. In its final form, it takes the form of the following three layers: the first being the yellowish straw-colored layer that contains cellular plasma; the second layer consisting of the fibrin clot; and a redder third layer that contains red blood cells. After separating the red blood cells and plasma, the fibrin clot (PRF) was collected. More significantly, because it contains high concentrations of platelets, the intersecting layer between fibrin and cellular plasma was preserved meticulously.

Surgical treatment protocol

**Figure 1:**
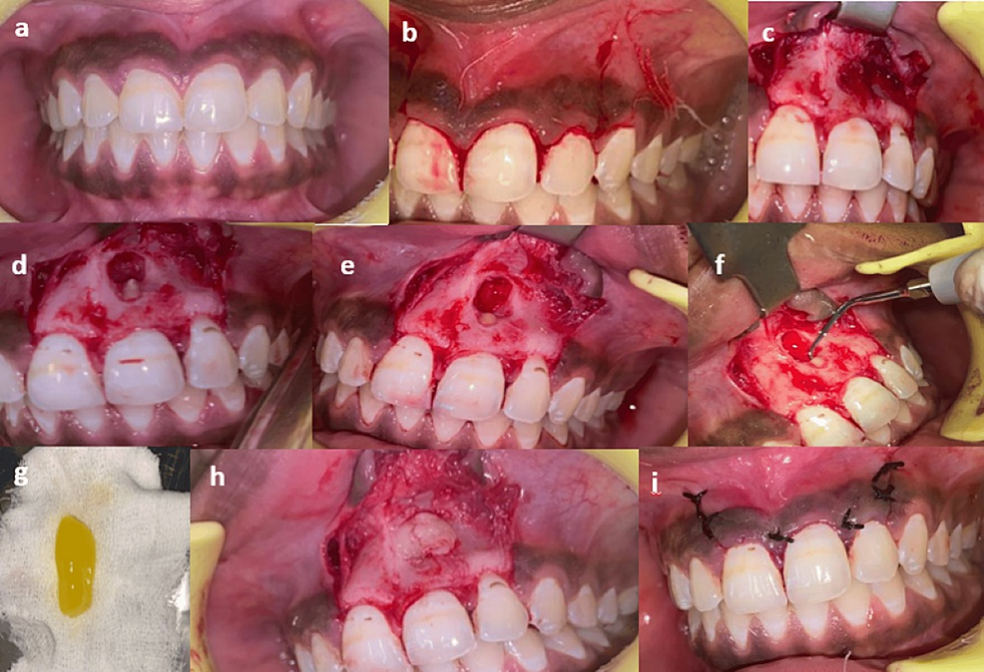
(a) Preoperative image. (b) Incision made from the distal of 11 to the distal of 22. (c) Reflection of the full-thickness mucoperiosteal flap. (d) After cyst enucleation. (e) Root end resection. (f) Root end preparation. (g) Advanced platelet-rich fibrin (PFR). (h) Placement of PRF in the cavity after root end filling done using Biodentine. (i) Immediate postoperative image.

**Figure 2:**
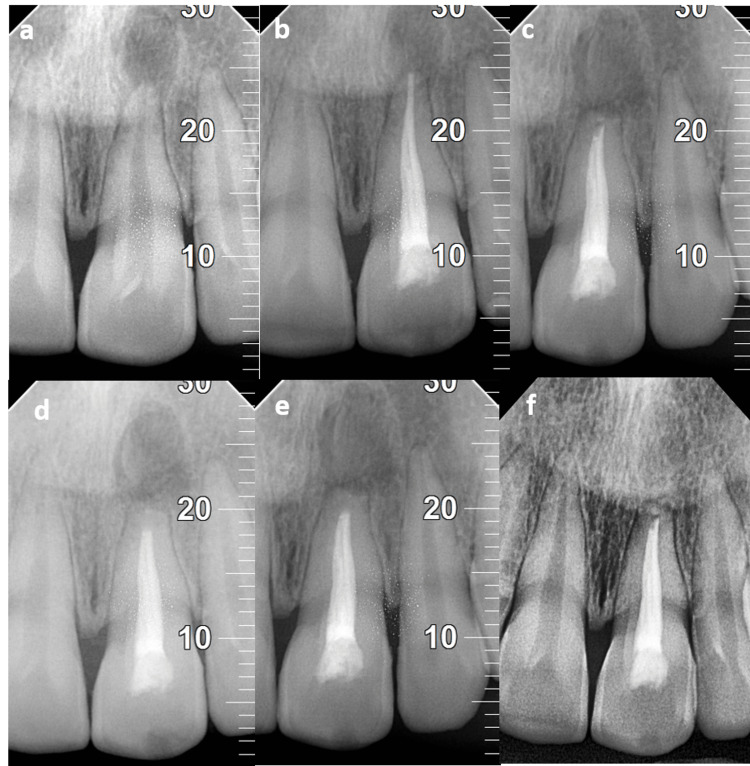
(a) Preoperative radiograph. (b) After root canal treatment. (c) Immediate postoperative radiograph. (d) Three-month follow-up. (e) Six-month follow-up. (f) 12-month follow-up. Markings (10, 20, 30): scale indicating the length and extent of the lesion. It also indicates that the lesion has subsided periodically, as is evident by the measurement.

After local anesthesia administration in the maxillary anterior region using lidocaine with adrenaline, a full-thickness mucoperiosteal flap was elevated on the buccal aspect and then a cyst was enucleated and the retrograde filling was performed surgically. A biopsy was obtained for examination by a pathologist. Following apicoectomy and root-end preparation, Biodentine (Septodont, USA) was used for the retrograde filling. After the PRF was positioned inside the cystic cavity, non-resorbable sutures were placed which were removed after 10 days. Upon examination of the biopsy obtained during the surgical procedure, a periapical cyst was verified. Subsequently, the patient underwent an evaluation at three-, six, and nine-month follow-ups. During the review period, no signs of tenderness, discomfort, or inflammation were noted. During the follow-up visits, regular clinical examinations and professional oral hygiene aids were provided, as shown in Figures 1a-1i. The radiographic images are presented in Figures 2a-2f.

## Discussion

Periapical cysts form gradually when an inflammatory process stimulates Malassez’s epithelial cell rests, and cystic fluid containing cholesterol crystals develops promptly surrounding the apex. Expansion of cysts can occur as a result of an increase in cystic fluid or infection [7]. Cysts account for 15% of all periapical lesions, and periapical cysts are remarkably the more frequent type accounting for 52.3-70.7% of all odontogenic cysts [8].

The treatment option is determined by many factors, including the lesion’s origin and extent, its association with vital structures, clinical characteristics, systemic status, and compliance of the patient [9]. The therapeutic protocol for such types of cysts continues to be a source of debate and contention. Endodontic therapy is a common conservative treatment option for small lesions among dentists. In our situation, multiple visits of endodontic treatment were done with intracanal medication using calcium hydroxide to reduce microbial levels. Calcium hydroxide dressings serve as a supplementary aid to instrumentation and irrigation solutions in decreasing bacterial load leading to a more effective disinfection in areas such as ramifications and dentinal tubules. The hygroscopic properties of calcium hydroxide are highly effective in reducing exudate in clinical settings. According to previous research, calcium hydroxide medication must be applied for at least two weeks to have an effective antimicrobial action [10]. However, in the case of large-sized periapical lesions, conservative root canal treatment will not completely eradicate that bacteria, so marsupialization/decompression or even enucleation can be performed [11]. An epoxy resin-based sealer, i.e., AH Plus®, was selected as it has greater antimicrobial activity against endodontic pathogens than other root canal sealers available. PRF, alongside promoting healing, has been shown to decrease postoperative hematomas owing to its affirmative sealing capability with fibrin adhesive [12]. The successful outcome of PRF application depends exclusively on the time required for the collection of blood and immediate transfer to the centrifugation machine. When blood comes in proximity to non-anticoagulant tubes, it coagulates, and centrifugation only takes a short time to extract concentrated fibrinogen from the tube’s middle and upper parts [13]. The only practical way to acquire a clinically useful PRF is by immediate manipulation. Longer centrifuging and blood collection times will render the process ineffective. The tube presents with fibrin showing diffuse polymerization which will result in the production of a small amount of fibrinogen lacking uniformity [14]. When used as a retrograde filling material, Biodentine demonstrated significantly higher sealing capability than mineral trioxide aggregate (MTA) and intermediate restorative material [15]. According to a systemic review conducted in 2020, Biodentine has a higher sealing ability than MTA during the first 24 hours, but both materials are equal after one week [16]. Regarding mechanical properties, Biodentine outperforms MTA in clinical applications. Furthermore, Biodentine has shown reduced time setting and ease of use but has poor radiopacity which limits the visualization of the retrograde obturation [17].

## Conclusions

The clinical success of PRF in the surgical management of radicular cysts demonstrates that, during periapical surgery, it can be used as a healing biomaterial due to its slow and sustained release of key growth factors, causing the membrane to promote the environment for an extended period in the course of remodeling. As a result, clinicians should acknowledge the advantages of PRF and incorporate it into various treatment modalities to provide patients with a more economical option.
